# Evaluating mucus exudation dynamics through isotopic enrichment and turnover of skin mucus fractions in a marine fish model

**DOI:** 10.1093/conphys/coaa095

**Published:** 2020-12-01

**Authors:** Borja Ordóñez-Grande, Laura Fernández-Alacid, Ignasi Sanahuja, Sergio Sánchez-Nuño, Jaume Fernández-Borràs, Josefina Blasco, Antoni Ibarz

**Affiliations:** Department of Cell Biology, Physiology and Immunology, University of Barcelona, Barcelona, Spain

**Keywords:** exudation, isotopic natural abundance, mucus renewal, Sparus aurata, skin mucus fractions

## Abstract

Fish skin mucus is composed of insoluble components, which form the physical barrier, and soluble components, which are key for interrelationship functions. Mucus is continuously secreted, but rates of production and exudation are still unknown, as are the underlying mechanisms. Using stable isotope analysis, here, we evaluate skin mucus turnover and renewal in gilthead sea bream, separating raw mucus and its soluble and insoluble fractions. Isotopic abundance analysis reveals no differences between mucus and white muscle, thus confirming mucus samples as reliable non-invasive biomarkers. Mucus production was evaluated using a single labelled meal packaged in a gelatine capsule, with both ^13^C and ^15^N, via a time-course trial. ^13^C was gradually allocated to skin mucus fractions over the first 12 h and was significantly (4-fold) higher in the soluble fraction, indicating a higher turnover of soluble mucus components that are continuously produced and supplied. ^15^N was also gradually allocated to mucus, indicating incorporation of new proteins containing the labelled dietary amino acids, but with no differences between fractions. When existent mucus was removed, dietary stable isotopes revealed stimulated mucus neoformation dependent on the components. All this is novel knowledge concerning skin mucus dynamics and turnover in fish and could offer interesting non-invasive approaches to the use of skin mucus production in ecological or applied biological studies such as climate change effects, human impact, alterations in trophic networks or habitat degradation, especially of wild-captured species or protected species.

## Teaser

Isotopic signature of fish skin mucus represents an advantageous non-invasive way to study fish ecology and applied biology. Our results demonstrate that stable isotope enrichment in skin mucus is modified by one meal, and the rate of isotope allocation depends on the soluble or insoluble components of the mucus.

## Introduction

Stable isotope analysis (hereafter SIA) is a very powerful and effective tool to determine trophic relationships, dietary switching and migrating patterns when studying fish ecology (Maruyama et al., 2001; Church et al., 2009). SIA has been used to evaluate dietary sources and the trophic position of fish. From a productive point of view, SIA has also been used to trace the metabolic fate of food nutrients and their distribution within fish tissues, given different dietary sources, regimes or rearing conditions (Beltrán et al., 2009; Felip et al., 2012, 2015; Martín-Pérez et al., 2013). Irrespective of the aim of those studies, in traditional methods of isotope analysis, fish must be killed in order to sample the tissues most commonly used: the white muscle and liver (Logan et al., 2006; Guelinckx et al., 2007; Boecklen et al., 2011). Dorsal white muscle is considered the best tissue as it represents fish dietary adaptation isotopically (Martín-Pérez et al., 2013; Busst et al., 2015; Vander Zanden et al., 2015). Meanwhile, the liver, blood and plasma exhibit shorter half-lives than dorsal muscle (Thomas and Crowther, 2015; Vander Zanden et al., 2015). The use of fish tissue samples necessarily implies invasive or fatal collection methods. To avoid this, non-invasive collection of alternative tissues, such as fin and scales, is increasingly used (Busst et al., 2015; Busst and Britton, 2016). However, early experiments reported that the isotopic half-lives exhibited by these tissues can be longer than those of dorsal muscle (Busst and Britton, 2018; Winter et al., 2019). A recently proposed and encouraging alternative for isotopic analysis is to use skin mucus. Although limited SIA has been performed on fish mucus, and mostly in freshwater fish species, initial suggestions are that mucus has a relatively fast turnover, similar to or faster than that of muscle (Church et al., 2009; Maruyama et al., 2015, 2017; Shigeta et al., 2017; Winter et al., 2019).

The importance of skin mucus for fish physiology and welfare studies has therefore increased over the past decade. As the most external bodily layer positioned between the epidermis and the environment, fish skin mucus provides a protective barrier against physical, mechanical and chemical agents as well as both biotic and abiotic stressors (reviewed in Esteban, 2012). Skin mucus is produced mainly by goblet cells located in the epithelium and composed mainly by water and gel-forming macromolecules such as mucins and other glycoproteins (Ingram, 1980; Sheppard, 1994). Nevertheless, some components are incorporated via the secondary circulatory system and the epithelial cells themselves (Easy and Ross, 2009). Most of the components of skin mucus are related to mucus defences (Rajan et al., 2011; Sanahuja and Ibarz, 2015; Cordero et al., 2015; Patel and Brinchmann, 2017; Pérez-Sánchez et al., 2017, Sanahuja et al., 2019a,b), to mucus metabolites such as glucose or lactate, or to hormones like cortisol (Guardiola et al., 2016; Fernández-Alacid et al., 2018, 2019a,b). Mucins can generally be considered the insoluble components, or the insoluble fraction, of the mucus that provide the physico-chemical properties on which the biological functions depend. Mucus viscosity is a property that is mainly attributed to mucin contents and hydration, and it provides the surface of the body of the fish with rheological, viscoelastic or adhesive characteristics (Fernández-Alacid et al., 2018, 2019b). The soluble components, or soluble fraction, come from goblet cells as well as from epithelial cells and the inner body; they endow the mucus with its protective, structural and metabolic properties (Cordero et al., 2015; Sanahuja and Ibarz, 2015; Sanahuja et al., 2019a,b; Fernández-Alacid et al., 2018, 2019a). Moreover, skin mucus is continuously secreted and replaced to prevent pathogen adhesion (Benhamed et al., 2014), but this production and secretion can be augmented in response to external factors such as stress by increasing skin mucous cell number or size (Sheppard, 1994; Vatsos et al., 2010; Fernández-Alacid et al., 2018). Recently, we proposed a methodology to study mucus dynamics via stable isotope enrichment from one force-fed meal (Ibarz et al., 2019), following the methods proposed by Beltrán *et al.* (2009) and Felip *et al.* (2012, 2015) to study the fate of ingesta. However, no studies have yet addressed exudation dynamics of each mucus fraction, soluble and insoluble, considering their different functions and putatively different internal origin.

To fill some of the gaps that still exist in our knowledge of fish skin mucus as a bioindicator, in this study, we used SIA and experimental procedures on the gilthead sea bream, *Sparus aurata,* fish model. Specifically, our objectives were as follows: (i) to determine the isotopic signature (for the isotopes 15 N and 13C) of skin mucus, for the first time analysing soluble and insoluble mucus fractions, comparing these with other tissues such as the liver, white muscle and plasma; (ii) to determine the new mucus production via the isotopic enrichment (δ15N and δ13C) of the total and mucus fractions after one force-fed meal; and (iii) to test the effects of a renewal process (by removal of the existing mucus) on the mucus production via the isotopic enrichment. The SIA technique and procedures allowed us to determine which mucus components are more easily replaced and provided practical approaches to the study of mucus production and renewal rates under different conditions, stimuli or challenges in ecological or applied biological studies.

## Materials and methods

### Animals

Sea bream juveniles were obtained from a local provider (Piscimar, Burriana, Spain) and acclimated indoors at the Faculty of Biology facilities (University of Barcelona, Barcelona, Spain) at 22°C for 1 month, using a standard commercial fish feed (Skretting ARC, Burgos, Spain). A total of 60 fishes (body weight average, 186.1 ± 5.31 g) were tagged with a passive integrated transponder (Trovan Electronic Identification Systems, UK) and fed twice a day a daily ration of 1.5% of body weight. The rearing systems were controlled and monitored as described in Ibarz *et al.* (2019). All animal handling was conducted following the European Union Council (86/609/EU) and Spanish national and Catalan regional norms and procedures, with approval from the University of Barcelona Ethics and Animal Care Committee (permit no. DAAM 9383).

### Time-course enrichment trial

Two different SIA trials were conducted. The first included a time-course enrichment trial of skin mucus and representative tissues together with study of the natural isotopic abundances. We used 50 fishes to perform the time-course isotopic enrichment via skin mucus exudation. In accordance with previous studies on the use of the fate of dietary nutrients in gilthead sea bream, the meal was labelled with algal starch (3% ^13^C) and algal protein (1% ^15^N) (Beltrán et al., 2009; Felip et al., 2013). The feed was ground, mixed with the labelled compounds and packed into gelatine capsules (PsoriasisEX Ltd, Germany) following the method of Ibarz *et al.* (2019). Four sampling points were scheduled at 0, 6, 12 and 24 h after feeding, and 10 fishes were sampled at each point. The fishes were lightly anesthetized (0.1 g.L^−1^ MS-222) force-fed four gelatine capsules of approximate 0.2 ml each, using a gastric cannula containing a meal equivalent to 0.6% of body weight. To determine the natural abundance of ^13^C and ^15^N in tissue and skin mucus, 10 fishes received the same diet and meal weight but containing similar proportions of unlabelled protein and starch. These fishes were sampled as 0 h after feeding, to determine isotopic signature. To obtain the diet isotopic signature, three independent samples of the unlabelled diet were used.

### Renewal trial

The second SIA trial was the renewal trial aiming to analyse the skin mucus isotopic renewal by previous mucus removal. We used 10 fishes that were slightly anesthetized and had mucus removed after drying their body surface with absorbent sterile paper for few seconds (4–5 s) and then they were immediately force fed, as described above, to be further sampled at 24 h post-feeding. As control fish, animals sampled at 24 h of time-course trial were used.

### Sample collection

After force feeding, the fishes were held for a minute in individual tanks to check regurgitation and to ensure recovery before being returned to their rearing tank. In the time-course enrichment trial, after being anesthetized, mucus samples were immediately collected as described in Fernández-Alacid *et al.* (2018) and in Ibarz *et al.* (2019). Briefly, a sterile glass slide was used to carefully remove mucus from the over-lateral line, starting from the front and sliding in the caudal direction. The glass was gently slid along both sides of the animal only three times, to avoid epithelial cell contamination (Fernández-Alacid et al., 2018), and the skin mucus was carefully pushed into a sterile tube (1.5 ml) and stored at −80°C until analysis. The non-desirable operculum, ventral-anal and caudal fin areas were avoided. Afterwards, the fishes were weighed and laterally photographed to record the mucus extraction area and killed by severing the spinal cord, and the plasma, liver and muscle were sampled to measure stable isotope enrichment. To verify the post-prandial process, plasma glycaemia was analysed measuring plasma glucose using a commercial kit (Spinreact, Spain) adapted to 96-well microplates. In the renewal trial, after being anesthetized, mucus samples were collected 24 h post-prandial, as described above, and the fishes were weighed and laterally photographed to record the area the mucus was collected from. All fish images were analysed using ImageJ software manually delimiting the mucus extraction area for each individual fish and using our own software (Schindelin et al., 2015) to calculate the skin area (in cm^2^) and the corresponding mucus production (as mg of mucus per cm^2^).

### δ^15^N and δ^13^C tissue determination

Skin mucus samples were lightly homogenized using a sterile Teflon implement to avoid possible depositions on the bottom of the tube. For each sampling point and the blanks, five mucus samples were used to measure the total raw mucus isotopic abundance or enrichment, and five different samples were used to measure the insoluble and soluble mucus fractions. To obtain mucus fractions, raw samples were centrifuged at 14 000 g as described in Fernández-Alacid *et al.* (2018) to separate the insoluble (pellet) and soluble components. For the post-prandial trial, the pieces of the liver (100 mg) and white muscle (300 mg) were ground in liquid N_2_ using a pestle and mortar to obtain a fine powder. Mucus samples, plasma and tissue samples were dried using a vacuum system (Speed Vac Plus AR, Savant Speed Vac Systems, South San Francisco, CA, USA). Pre-weighed vials were used to dry the insoluble and soluble mucus fractions and to calculate water content. Dried aliquots ranging from 0.3 to 0.6 mg were accurately weighed in small tin capsules (3.3–5 mm, Cromlab, Barcelona, Spain) and analysed for their C and N isotope composition using a Mat Delta C isotope-ratio mass spectrometer (IRMS, Finnigan MAT, Bremen, Germany) coupled to a Flash 1112 Elemental Analyser (ThermoFisher Scientific, Madrid, Spain); both at the Scientific Services of the University of Barcelona: CCiTUB. The EA-IRMS burned the samples and converted them into gas (N_2_ and CO_2_), and then transported them through a continuous helium flux to determine the percentage carbon and nitrogen content in the samples. Isotope ratios (^13^C/^12^C and ^15^N/^14^N) in the samples were expressed on a relative scale as deviation, referred to in delta (δ) units (parts per thousand, ‰) and according to the international standards: PDB (Pee Dee Belemnite, a calcium carbonate) for C and air for N.

The net enrichment of tissue or atom percentage excess (APE) was calculated from the difference between the at.% and their corresponding blank at.% values:

$$ \mathrm{APE}=\mathrm{at}.\%\ \mathrm{sample}\hbox{--} \mathrm{at}.\% \ \mathrm{blank}$$

Finally, the results for total allocation were expressed as a percentage of ingested dose in each tissue (^13^C or ^15^N g/100 g of ^13^C or ^15^N ingested) using APE, molecular weight and Avogadro’s number:

$$\begin{align*} &100\cdotp \Big(\left({\mathrm{g}}^{13}\mathrm{C}\ {\mathrm{or}}^{15}\mathrm{N}/\mathrm{g}\ \mathrm{m}.\mathrm{fr}.\right)\cdotp \left(\mathrm{g}\ \mathrm{m}.\mathrm{fr}./\mathrm{g}\ \mathrm{tissue}\right)\\ &\qquad\cdotp \left(\mathrm{g}\ \mathrm{tissue}/\mathrm{g}\ \mathrm{b}.\mathrm{w}.\right)/\left(\mathrm{g}\ {\mathrm{ingested}}^{13}\mathrm{C}\ {\mathrm{or}}^{15}\mathrm{N}/\mathrm{g}\ \mathrm{b}.\mathrm{w}.\right)\Big) \end{align*}$$

where m.fr. is the mucus fraction and b.w. is body weight. Tissue values for white muscle and plasma were obtained according to the literature (Felip et al., 2013 and Fazio et al., 2013, respectively). For skin mucus, total exudation of mucus was referred to extraction area in cm^2^ and in cm^2^ per g of fish.

### Statistical analysis

For the comparison of the isotopic signature between diet, mucus and tissues, one-way analysis of variance (ANOVA) was performed. For the time-course enrichment trial, statistical differences in isotopic enrichment throughout the post-prandial samples were analysed by one-way ANOVA. For the renewal trial, the comparison between the 24 h renewal enrichment and 24 h time-course enrichment was performed using Student’s *t*-test. For all the statistical analysis, a previous study of homogeneity of variance was performed using Levene’s test. When homogeneity existed, Tukey’s *post-hoc* test was applied, whereas if homogeneity was not established, the T3-Dunnet test was applied. All statistical analysis was undertaken using SPSS for Windows, v22.0 (IBM Corp, Armonk, NY, USA), and all differences were considered statistically significant at *P* < 0.05.

## Results

### Isotopic signature

**Figure 1:**
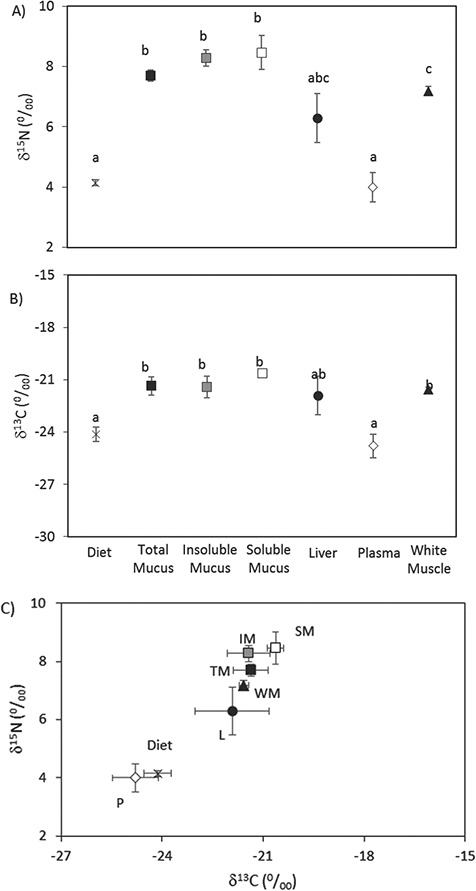
Stable isotope abundances ^13^C (A) and ^15^N (B), and a biplot of the isotopic signature (C). Values are means ± s.e.m. of five individual samples for total mucus (TM), insoluble mucus (IM), soluble mucus (SM), liver (L), plasma (P) and white muscle (WM). For dietary isotopic abundance, three independent samples were analysed. Different letters indicate significant differences between mucus fractions, tissues or diet (*P* < 0.05, ANOVA and the *post-hoc* Tukey test).

The stable isotope abundances (δ^15^N and δ^13^C) for diet and each tissue analysed, as well as the isotopic signature (biplot δ^15^N vs δ^13^C), are shown in Fig. 1. Diet δ^15^N and δ^13^C were 4.2 ± 0.2‰ and −24.1 ± 0.4‰, respectively. The isotopic composition of tissues, at 1 month of diet acclimation, showed that both ^15^N abundance and ^13^C abundance depends on the tissue studied. For δ^15^N, total mucus and both its fractions (soluble and insoluble) had values around 8‰, with no differences (*P* > 0.05), while white muscle values were significantly lower: 7.2 ± 0.4‰. The liver showed intermediate δ^15^N values, between the low diet values and the high ones for mucus or white muscle, whereas plasma values were equivalent to those of the diet. For δ^13^C, mucus and white muscle ranged from −22‰ to −20‰: significantly higher than for diet, whereas the liver and plasma values matched those of the diet.

### Time-course trial

**Figure 2:**
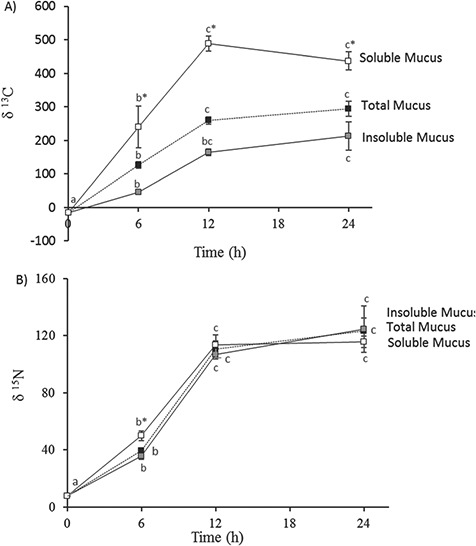
Time course of mucus isotopic enrichment after a single meal: (A) δ^13^C levels and (B) δ^15^N levels. Values are means ± s.e.m. of the five individual samples. Different letters indicate significant differences during the time course (*P* < 0.05, ANOVA and the *post-hoc* Tukey test) and * indicates significant differences between soluble and insoluble fractions (*P* < 0.05, Student’s *t*-test).

**Figure 3:**
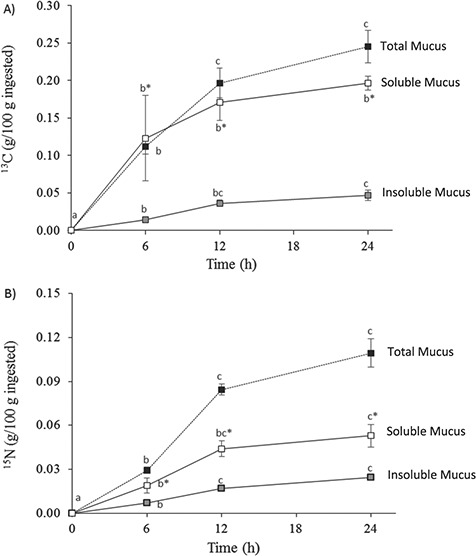
Time course of total isotopic allocation to mucus fractions after a single meal: (A) ^13^C levels and (B) ^15^N levels. Values are means ± s.e.m. of five individual samples. Total allocation, expressed as percentage (g/100 g of ingesta), was calculated as indicated above in M&M. Different letters indicate significant differences during the time course (*P* < 0.05, ANOVA and the *post-hoc* Tukey test) and * indicates significant differences between soluble and insoluble fractions (*P* < 0.05, Student’s *t*-test).

SIA was used to determine the incorporation of the isotopes into the mucus fractions (soluble and insoluble) after force feeding the fish with a labelled meal. Figures 2 and 3 show isotope enrichment values with respect to total ingested stable isotopes, respectively, over the one-day time-course trial (0 h, 6 h, 12 h and 24 h after feeding). The stable isotope enrichment (Fig. 2A) revealed that the soluble fraction of mucus, SM, incorporated more ^13^C than the insoluble fraction, IM, did: delta values were 5-fold higher at 6 h (240 ± 55 vs 45 ± 5 ‰, *P* < 0.05) and remained over 3-fold higher at 12 h (489 ± 15 vs 165 ± 9 ‰, *P* < 0.05). The time interval between 12 h and 24 h after feeding saw no further ^13^C enrichment. Surprisingly, ^15^N was not incorporated differently into SM and IM, with values matching those for total mucus (Fig. 2B). However, at 6 h, a slightly higher enrichment into SM was detected (*P* < 0.05). The time-course trial also showed that after 12 h, most ^15^N enrichment had occurred, with no significant increase between 12 h and 24 h. This demonstrated that maximum enrichment of labelled nutrients (both ^15^N and ^13^C) into mucus components after a single meal is achieved before 12 h has passed.

To calculate the total ^13^C and ^15^N enrichment into each mucus fraction, SM and IM percentages were obtained gravimetrically. No differences between percentages in the fractions were detected during the time-course samplings and the means obtained were 82.4 ± 2.1% for the soluble fraction and 15.3 ± 1.6% for the insoluble fraction. Correspondingly, when isotope allocation was expressed as total isotope ingested (Fig. 3), our data showed that the soluble fraction was highly labelled (*P* < 0.05) for both isotopes than the insoluble fraction was. The ingested ^13^C (Fig. 3A) sent to the raw (or total) mucus gradually increased from 0 h to 12 h, and then increased slightly at 24 h to the maximum values of 0.25 ± 0.02%, with apparently faster enrichment (6 h) into SM and more gradually over the 24-h time interval for IM. In this way, the ingested ^15^N (Fig. 3B) destined for the raw mucus showed the highest enrichment from 6 h to 12 h, achieving a maximum of 0.11 ± 0.01% at 24 h. As opposed to ^13^C, total ^15^N incorporated into SM only doubled that incorporated into IM, although significantly at each time interval.

**Figure 4:**
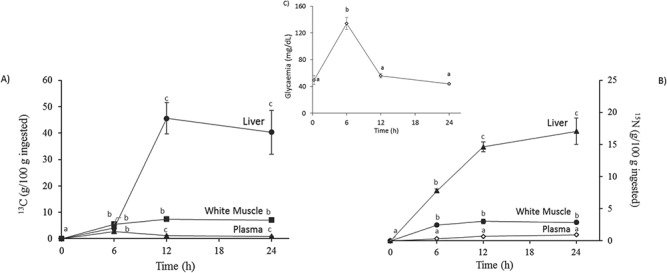
Time course of isotopic allocation in plasma, liver and white muscle for ^13^C (A) and ^15^N (B). Values are means ± s.e.m. of five individual samples. Total allocation, expressed as percentage (g/100 g of ingested), was calculated as indicated above in M&M. Post-prandial plasma glucose levels are also shown (C). Different letters indicate significant differences during the time course (*P* < 0.05, ANOVA and the *post-hoc* Tukey test).

Liver, muscle and plasma ^15^N and ^13^C enrichment was also calculated with respect to total isotope ingestion, as explained in M&M and represented in Fig. 4, to compare further with amounts incorporated into mucus and their dynamics. Contrary to the case of mucus enrichment, for each of the tissues studied (Fig. 4A), ^13^C was not incorporated gradually but with a peak in plasma and white muscle at 6 h, and with a marked increase in the liver between 6 h and 12 h, to values as high as over 40% of ingested ^13^C. This demonstrates considerable assimilation of the labelled meal when using the proposed gelatine capsule method. In the case of the fate of ^15^N, that ingested in a single meal was gradually incorporated between 0 and 12 h, reaching values of around 15% for the liver, 3% for white muscle and 1% for plasma. As confirmation of feed assimilation, plasma glucose was measured (Fig. 4C) and showed a post-prandial peak value at 6 h with a return to the expected basal values at 12 h and 24 h.

### Renewal trial

In the second experiment, the enrichment of stable isotopes into recently exuded (new) mucus was analysed using the same force-fed meal method. To this end, before feeding the gelatine capsules with the labelled meal to the fish, their skin mucus was individually removed and, to avoid healing so as to be able to collect mucus twice in a short time period, only 24 h sampling was performed. Table 1 summarizes data comparing mucus volume collected and isotopic enrichment between control samples (without previous mucus removal) and ‘renewal’ samples (24 h after the mucus removal). The mucus removal provoked a significant reduction in mucus collected (290 ± 35 mg per fish) with respect to control mucus (510 mg ± 49 mg per fish) as well as in the mucus exuded per skin area or per 100 g of fish, as there were no differences in fish collecting area or fish weight. Referring the fate of the ingested diet to the mucus renewal process, our data demonstrated that new mucus exuded in 24 h showed greater enrichment for ^13^C, which was doubled in total mucus (*P* < 0.05) and affected both soluble and insoluble mucus components. ^15^N enrichment of the new mucus also increased, although it was only significant for 30% enrichment of the SM. However, the volume of mucus collected was reduced, as mentioned above, which consequently affected the total isotope allocations in raw mucus and its fractions. Thus, the results we calculated of the fate of one ingested meal showed that labelled ^13^C in the new exuded mucus reached the control values in total mucus and IM but did not in SM. In contrast, labelled ^15^N did not reach control values in new total mucus or SM, evidencing that the mucus turnover differed according to the origin of each labelled dietary component, starch for ^13^C or protein for ^15^N, and even depending on SM or IM.

**Table 1:** Collected skin mucus and isotopic enrichment (at 24 h post-prandial) from control fish and renewal fish

	**Control**	**Renewal**
**Mucus exudation**		
*Collected mucus (mg)*	510 ± 49	290 ± 35^*^
*Mucus per area (mg/cm^2^)*	7.2 ± 0.7	4.1 ± 0.5^*^
*Mucus collected per fish (mg/100 g)*	231 ± 18	131 ± 15^*^
**Isotopic enrichment**		
^13^C enrichment (‰)		
*Total mucus*	294 ± 22	638 ± 16^*^
*Insoluble mucus*	213 ± 43	355 ± 27^*^
*Soluble mucus*	437 ± 27	578 ± 4^*^
^15^N enrichment (‰)		
*Total mucus*	124 ± 9	147 ± 8
*Insoluble mucus*	125 ± 16	149 ± 7
*Soluble mucus*	116 ± 4	149 ± 4^*^
^13^C (mg/100 g ingested)		
*Total mucus*	245 ± 21	251 ± 18
*Insoluble mucus*	47 ± 7	54 ± 5
*Soluble mucus*	196 ± 9	138 ± 14^*^
^15^N (mg/100 g ingested)		
*Total mucus*	110 ± 10	58 ± 10^*^
*Insoluble mucus*	24 ± 1	20 ± 2
*Soluble mucus*	53 ± 4	36 ± 2^*^

Values are expressed as mean ± s.e.m. *N* = 10 for data of mucus exuded and *n* = 5 for data of isotopic enrichment. ^*^ indicates significant differences between control and renewal groups (from Student’s *t*-test).

## Discussion

Most studies of fish skin mucus have been performed on the soluble fraction, considering isotopic composition (Church et al., 2009; Maruyama et al., 2017; Shigeta et al., 2017) or mucus properties (reviewed in Esteban, 2012 and in Benhamed et al., 2014). In the present work, we studied separately raw mucus and its soluble and insoluble fractions. We compared their natural isotopic signatures and isotopic enrichment after a force-fed meal or during a renewal process using SIA to gain better knowledge of the mechanisms underlying the rhythm of skin mucus exudation and the importance of its soluble and insoluble components.

The few previous SIA studies of fish mucus used skin mucus from defrosted fish directly wiped on glass microfiber filters (Maruyama et al., 2015, 2017; Shigeta et al., 2017; Winter et al., 2019). That would correspond to raw mucus collected in the current experiment, which we obtained directly from live animals. However, no studies have compared stable isotopes abundance in whole (raw) mucus and either its soluble (typically used to study mucus properties) or insoluble fraction (much less used: only to study physical properties such as viscosity). The first result derived from our analysis of mucus fractions was the am ount of each fraction in gilthead sea bream skin mucus. These amounts were around 80% for soluble fraction, and 20% for the insoluble fraction, irrespective of the moment and condition of sample collection in the current trials. From our best knowledge, no data have been published on mucus fraction amounts in fish species to compare with our results in sea bream. Due to recent findings that reported specific changes in mucus physical properties in response to stress conditions in pelagic species such as sea bream, sea bass and meagre (Fernández-Alacid et al., 2019a) and in benthonic species such as Senegalese sole (Fernández-Alacid et al., 2019b), more experiments are necessary to explain the role of mucus fractions better; for instance, in conditions that induce chronic mucus exudation or using hormonal implants to favour mucus exudation, in the same species or others.

In spite of the fraction amounts, the natural abundance of neither of the stable isotopes considered, ^13^C and ^15^N, differed between the soluble and insoluble fractions or compared with raw mucus. These data would indicate the equivalence of analysing the whole mucus with respect to the soluble fraction: the most common way to study other mucus properties in fish. In the current study, the differences between diet isotopic abundance and tissue isotopic abundances at 1 month of diet acclimation resulted in higher values: over 2.7‰ and 3.5‰ for ^13^C and ^15^N, respectively. In the literature, it has been reported that isotopic discrimination between predators and their prey increases as the protein quality decreases, especially for ^15^N (Roth and Hobson, 2000). In aquaculture conditions, higher isotopic values in tissues were attributed to plant material in the diet (Beltrán et al., 2009; Busst and Britton, 2016) as was also reported for whole mucus (Winter et al., 2019). The second finding derived from the natural abundance in mucus fractions under the current conditions confirmed that skin mucus in both forms, raw mucus or SM, collected from live fish provides the same information as white muscle: the classic tissue used to evaluate dietary trophic effects (Busst et al., 2015; Vander Zanden et al., 2015). Considering that mucus have a relatively fast turnover, as we discuss below, with an isotopic half-live similar to muscle (Church et al., 2009; Shigeta et al., 2017; Winter et al., 2019), together with the valuable less-invasive way to obtain fish samples; we consider mucus as a reliable alternative in aquatic stable isotope studies. Thus, the proposed procedure could also be useful in threatened species or in conservation studies, where fish sacrifice are inadvisable or prohibited, to evaluate the effects of environmental challenges, to known the fish status, the rearing conditions, etc.

SIA has successfully been used to study the metabolic fate of food nutrients (Hesslein et al., 1993; MacAvoy et al., 2005; Martín-Pérez et al., 2012; Xia et al., 2013; Felip et al., 2012, 2015), and we recently demonstrated that SIA was also valid for mucus studies, as skin mucus is also a fate of dietary components (Ibarz et al., 2019). In contrast to other SIA studies of mucus, data obtained in the current study provide information on the amount of isotope enrichment into raw mucus after 24 h for the first time: 0.25% and 0.1% of ingested ^13^C and ^15^N, respectively. Both plasma glucose and plasma ^13^C allocation showed the expected pattern of one marked peak 6 h post-prandial, in agreement with that reported by Felip *et al.* (2013, 2015) using a stable isotope post-feeding trial, or by Montoya *et al.* (2010) and Gómez-Milán *et al.* (2011), who analysed plasma glycaemia performance after ingesta. Moreover, both lower levels (<1%) of total stable isotope allocation per g of ingested isotopes confirmed that plasma does not act as a final fate but rather a transitory pathway with a fast turnover (Carter et al., 2019). ^15^N allocation to the liver also corresponded to reports in previous studies by Beltrán *et al.* (2009) and Felip *et al.* (2012, 2015) for gilthead sea bream after a force-fed meal. Interestingly, the improved method used for diet administration (Ibarz et al., 2019) corresponded to global higher levels of isotopic enrichment. In consequence, gelatine capsules filled with labelled diet would allow several precise checkpoints at which to measure the exact stable isotope dose ingestion, controlling any regurgitation event, and guaranteeing higher levels of label incorporation. This would be crucial for mucus studies, where lower levels of labelling are achieved.

The time course of allocation of each isotope to the mucus fractions provided relevant information on skin mucus formation and exudation processes. The allocation of ^13^C depended on the fraction analysed, being significantly higher for the soluble fraction. Unexpectedly we found that mucus fractions incorporated dietary ^15^N at the same rhythm, irrespective of whether to total mucus or the soluble or insoluble fractions. Whereas ^15^N enrichment is classically used as an indication of the origin of dietary protein, ^13^C is used as an indication of isotopic routing from several dietary constituents (protein, lipids and carbohydrates) (DeNiro and Epstein, 1977; Schwarcz and Schoeninger, 1991; Martín-Pérez et al., 2011). Thus, as could be expected, the great ^13^C enrichment and allocation to the soluble fraction of the mucus, composed of small molecules, would indicate a higher turnover of soluble metabolites than insoluble components, mainly mucopolysaccharides with slower synthesis rates. Interestingly, compared to isotope enrichment into tissues, mucus ^13^C enrichment was fast and continuous for the first 12 h, with maximum enrichment at 24 h. This is in contrast to plasma, where ^13^C allocation diminished after 12 h, and to both muscle and liver, with maximum incorporation at 12 h. These dynamics demonstrate that fish skin mucus not only represents the fate of dietary nutrients, as does muscle (Beltrán et al., 2009; Felip et al., 2013), but is continuously produced differently to the muscle or liver.

It is well accepted that insoluble components of all body mucosae are mainly mucins, which form mucus gel layers either directly or through their ectodomains, whereas soluble components are adhered or trapped within such layers (reviewed in Beck and Peatman, 2015). Thus, the key to understanding the different rhythms in isotope allocation demonstrated by the current results lies in the internal origin of the components of each fraction. Goblet cells located in the epithelium mostly exuded mucins and other heavy glycoproteins (Ingram, 1980), but their involvement in exuding soluble components is still not clear. Surprisingly, the appearance of ^15^N in skin mucus showed no differences between the soluble and insoluble components, with the amount of ^15^N g per 100 g of ^15^N ingested depending only on mucus fraction proportions. The rhythm of incorporation in mucus is also continuous and similar to that observed in the liver. Most of the ^15^N allocated to muscle is linked to new protein incorporation, and the lack of differences between soluble and insoluble components necessary implies that labelled dietary amino acids are incorporated at the same rhythm into both fractions, which has not previously been reported in the literature. Daily rhythms of mucus composition cannot be ruled out, as recently proposed by Lazado and Skov (2019) for several mucosal defences. Although the use of stable isotope enrichment via a single labelled meal would mask the daily rhythms of soluble and insoluble components of skin mucus, further studies should address both renewal rates and the daily/photoperiod rhythms of specific mucus components.

Other mucus components are presumably transferred from the circulatory system and the epithelial cells themselves (Easy and Ross, 2009). For instance, we recently demonstrated a high degree of correlation in some soluble components, such as glucose, lactate or cortisol, between a plasma overshot and a mucus overshot in response to stress (Fernández-Alacid et al., 2019a). Moreover, preliminary results reported by Reyes-López *et al.* (2019) suggest that skin cells provide skin mucus with a great number of soluble components. The results drawn from isotope ^13^C enrichment of soluble components seem to agree with the presence of such a secondary system of exudation and filtration of mucus components from plasma and epithelial cells. However, further studies using stable isotopes labelling will be necessary to understand the turnover of each specific mucus component better; for instance, by inducing mucus exudation with stress factors, as in Fernández-Alacid *et al.* (2018, 2019a), or with hormonal stimulation. Moreover, in a previous study of Senegalese sole, Fernández-Alacid *et al.* (2019b) demonstrated for the first time that mucus metabolite exudation could be side dependent in flatfish species with marked body asymmetry. In view of the present results on mucus secretion dynamics, the need for further studies on morphometrics and the distribution of mucus-secreting cells acquires greater importance to overcome the weakness of single-disciplinary approaches. It is known that goblet cell number can vary among different body regions of fish. Several studies have already shown that mucus cell distribution and skin gene expression vary in different fish skin areas, depending on species (brown trout and char, Pickering, 1974; cod, Caipang, et al., 2011; Atlantic salmon, Pittman et al., 2013; gilthead sea bream, Cordero et al., 2017; lumpfish, Patel et al., 2019; Senegalese sole, Fernández-Alacid et al., 2019b). New and complementary studies of the distribution of mucus cells and their underlying secretory mechanisms must be developed, for instance by combining SIA model studies with the histological approach both of which reinforce the idea of mucosa tissue. As the exudation and renewal rates of soluble and insoluble mucus fractions seem to be different, such studies would clarify the role of the diverse mucus cells in producing soluble and insoluble mucus components: the goblet cells, as the most abundant in all fish epidermal surfaces producing neutral mucus granules (Sheppard, 1993); sacciform cells and acidophilic granular cells, the latter producing basic proteins (Zaccone et al., 2001); and club cells, which secrete larger proteinaceous and smaller carbohydrate components (Faluso et al., 1993; Zaccone et al., 2001).

The aim of our current second trial was to evaluate the production and exudation of ‘new mucus’ by removing existent mucus. In this study, we demonstrate the presence of new exuded mucus by measuring the volume of the collected mucus (in mg per fish) and the turnover rate of new mucus via stable isotope enrichment, compared with unremoved mucus turnover. To the best of our knowledge, no similar experiment has been reported previously. In this way, we found half the volume of post-removal mucus after 24 h, compared to the amounts of natural, non-stressed, mucus collected. These results show that the biological barrier afforded by the mucus layer is compromised by any aquaculture handling processes, which exposes fish to mucus losses (weight classification, manual vaccination, high density, holding facilities, etc.). In specific conditions where mucus layers are shed or digested, pathogens can adhere to cells on the epithelium surface before mucus has been renewed (Cone, 2009; Benhamed et al., 2014). In contrast, in stressful situations, one of the most evident fish responses is an increase of skin mucus exudation (Shephard, 1994; Vatsos et al., 2010; Fernández-Alacid et al., 2018, 2019a,b). However, greater mucus exudation would modify the protein turnover in goblet mucus cells, which affected protein exudation in sea bass (Azeredo et al., 2015), reduced the total protein content in soluble mucus in sea bream (Fernández-Alacid et al., 2018) and Senegalese sole (Fernández-Alacid et al., 2019b) and even altered the mucus viscosity in Senegalese sole (Fernández-Alacid *et al.,* 2019b).

From a physiological point of view, isotope enrichment values allow us to determine the turnover modulation of mucus exudation via the incorporation of dietary components. Here, we have demonstrated that ^13^C enrichment of renewed mucus is higher than in control mucus (without previous removal), irrespective of the mucus fraction studied. These results indicate stimulated enrichment of ^13^C from dietary labelled starch, which necessarily implies an increase in intermediary metabolism to produce newly synthetized mucus components. Meanwhile, the new mucus exuded only saw ^15^N increased by 10%–20%: only significantly for soluble mucus components, thus illustrating a different dynamic from that of ^13^C. Only the protein fraction is labelled with ^15^N, whereas many other molecules labelled with ^13^C are incorporated into different tissue fractions (protein, carbohydrate, lipids). Although no data exist on mucus, other studies validated the stimulated turnover in tissues, for instance, under exercise conditions (Felip et al., 2012, 2013), where ^13^C turnover increased in the liver and ^15^N was in white muscle. Therefore, our current results suggest that when an external factor induces the formation of new mucus, we must take into account the different dynamics of each component during mucus neoformation, shown here by the different isotope enrichment. Thus, this SIA methodology is again proving to be a very interesting tool to study the turnover of mucus components and opens a new window for practical approaches to studying mucus production rates under different conditions, stimuli or challenges.

In summary, we conclude that our comparison of isotopic signature among mucus fractions and tissues confirms that mucus samples represent an advantageous less-invasive way to study fish ecology and applied biology. ^13^C and ^15^N allocation to skin mucus fractions was gradually achieved over the first 12 h post-feeding, but continuous until 24 h post-feeding, as opposed to what occurred in other tissues. The study of mucus fractions demonstrated that soluble components contained more ^13^C-labelled components than insoluble components, but no differences were shown in ^15^N, which exclusively marked newly synthetized proteins. Knowledge of these rhythms could be of great interest, considering that skin mucus one of the fates for the dietary additives (reviewed in Lee, 2015). When mucus renewal was induced by the removal of existent mucus, 24 h was not enough to achieve the non-stressed amount of mucus secretion, but via isotopic enrichment this replacement mucus showed a higher presence of de novo components. All these data on skin mucus exudation turnover in fish allow us to propose this methodology to improve knowledge via further fish studies of mucus turnover.

## References

[ref1] Azeredo R, Pérez-Sánchez J, Sitjà-Bobadilla A, Fouz B, Tort L, Aragão C, Oliva-Teles A , Costas B (2015) European sea bass (*Dicentrarchus labrax*) immune status and disease resistance are impaired by arginine dietary supplementation. PLoS One 10: 1–19. 10.1371/journal.pone.0139967 PMC4598043 26447480

[ref2] Beck BH, Peatman E (2015) Mucosal Health in Aquaculture. Elsevier Inc., Oxford, pp. 1–395

[ref3] Beltrán M, Fernández-Borrás J, Médale F, Pérez-Sánchez J, Kaushik S, Blasco J (2009) Natural abundance of 15N and 13C in fish tissues and the use of stable isotopes as dietary protein tracers in rainbow trout and gilthead sea bream. Aquac Nutr 15: 9–18.

[ref4] Benhamed S, Guardiola FA, Mars M, Esteban MÁ (2014) Pathogen bacteria adhesion to skin mucus of fishes. Vet Microbiol 171: 1–12. 24709124 10.1016/j.vetmic.2014.03.008

[ref5] Boecklen WJ, Yarnes CT, Cook BA, James AC (2011) On the use of stable isotopes in trophic ecology. Annu Rev Ecol Evol Syst 42: 411–440.

[ref6] Busst GMA, Bašic T, Britton JR (2015) Stable isotope signatures and trophic-step fractionation factors of fish tissues collected as non-lethal surrogates of dorsal muscle. Rapid Commun Mass Spectrom 29: 1535–1544. 26212169 10.1002/rcm.7247

[ref7] Busst GMA, Britton JR (2016) High variability in stable isotope diet-tissue discrimination factors of two omnivorous freshwater fishes in controlled ex situ conditions. J Exp Biol 219: 1060–1068. 26896544 10.1242/jeb.137380

[ref8] Busst GMA, Britton JR (2018) Tissue-specific turnover rates of the nitrogen stable isotope as functions of time and growth in a cyprinid fish. Hydrobiologia 805: 49–60.

[ref9] Caipang CMA, Lazado CC, Brinchmann MF, Rombout JHWM, Kiron V (2011) Differential expression of immune and stress genes in the skin of Atlantic cod (*Gadus morhua*). Comp Biochem Physiol D Genom Proteom 6: 158–162. 10.1016/j.cbd.2011.01.001 21262593

[ref10] Carter WA, Bauchinger U, McWilliams SR (2019) The importance of isotopic turnover for understanding key aspects of animal ecology and nutrition. Diversity 11: 84.

[ref11] Church MR, Ebersole JL, Rensmeyer KM, Couture RB, Barrows FT, Noakes DLG (2009) Mucus: a new tissue fraction for rapid determination of fish diet switching using stable isotope analysis. Can J Fish Aquat Sci 66: 1–5.

[ref12] Cone RA (2009) Barrier properties of mucus. Adv Drug Deliv Rev 61: 75–85. 19135107 10.1016/j.addr.2008.09.008

[ref13] Cordero H, Brinchmann MF, Cuesta A, Meseguer J, Esteban MA (2015) Skin mucus proteome map of European sea bass (*Dicentrarchus labrax*). Proteomics 15: 4007–4020. 26376207 10.1002/pmic.201500120

[ref14] Cordero H, Ceballos-Francisco D, Cuesta A, Esteban MÁ (2017) Dorso-ventral skin characterization of the farmed fish gilthead seabream (*Sparus aurata*). PLoS One 12: e0180438. 10.1371/journal.pone.0180438 PMC5493399 28666033

[ref15] DeNiro MJ, Epstein S (1977) Mechanism of carbon isotope fractionation associated with lipid synthesis. Science 197: 261–263. 327543 10.1126/science.327543

[ref16] Easy RH, Ross NW (2009) Changes in Atlantic salmon (*Salmo salar*) epidermal mucus protein compo sition profiles following infection with sea lice (*Lepeophtheirus salmonis*). Comp Biochem Physiol D Genom Proteom 4: 159–167. 10.1016/j.cbd.2009.02.001 20403764

[ref17] Esteban MÁ (2012) An overview of the immunological defenses in fish skin. ISRN Immunol 2012: 1–29.

[ref18] Faluso S, Tagliafierro G, Contini A, Ainis L, Ricca MB, Yanaihara N, Zaccone G (1993) Ectopic expression of bioactive peptides and serotonin in the sacciform gland cells of teleost skin. Arch Histol Cytol 56: 117–125. 8373656 10.1679/aohc.56.117

[ref19] Fazio F, Marafioti S, Arfuso F, Piccione G, Faggio C (2013) Comparative study of the biochemical and haematological parameters of four wild Tyrrhenian fish species. Vet Med 58: 576–581.

[ref20] Felip O, Ibarz A, Fernández-Borràs J, Beltrán M, Martín-Pérez M, Planas JV, Blasco J (2012) Tracing metabolic routes of dietary carbohydrate and protein in rainbow trout (*Oncorhynchus mykiss*) using stable isotopes ([13C]starch and [15N]protein): effects of gelatinisation of starches and sustained swimming. British J Nutr 107: 834–844. 10.1017/S0007114511003709 21806854

[ref21] Felip O, Blasco J, Ibarz A, Martin-Perez M, Fernández-Borràs J (2013) Beneficial effects of sustained activity on the use of dietary protein and carbohydrate traced with stable isotopes 15N and 13C in gilthead sea bream (*Sparus aurata*). J Comp Physiol B Biochem Syst Environ Physiol 183: 223–234. 10.1007/s00360-012-0703-6 22918602

[ref22] Felip O, Blasco J, Ibarz A, Martín-Pérez M, Fernández-Borràs J (2015) Diets labelled with 13C-starch and 15N-protein reveal daily rhythms of nutrient use in gilthead sea bream (*Sparus aurata*). Comp Biochem Physiol A Mol Integr Physiol 179: 95–103. 25263129 10.1016/j.cbpa.2014.09.016

[ref23] Fernández-Alacid L, Sanahuja I, Ordóñez-Grande B, Sánchez-Nuño S, Viscor G, Gisbert E, Herrera M (2018) Skin mucus metabolites in response to physiological challenges: a valuable non-invasive method to study teleost marine species. Sci Total Environ 644: 1323–1335. 30743845 10.1016/j.scitotenv.2018.07.083

[ref24] Fernández-Alacid L, Sanahuja I, Ordóñez-Grande B, Sánchez-Nuño S, Herrera M, Ibarz A (2019a) Skin mucus metabolites and cortisol in meagre fed acute stress-attenuating diets: correlations between plasma and mucus. Aquaculture 499: 185–194.

[ref25] Fernández-Alacid L, Sanahuja I, Ordóñez-Grande B, Sánchez-Nuño S, Herrera M, Ibarz A (2019b) Comparison between properties of dorsal and ventral skin mucus in Senegalese sole: response to an acute stress. Aquaculture 513: 734410.

[ref26] Gómez-Milán E, Haro C de, Sánchez-Muros MJ (2011) Annual variations of the plasmatic levels of glucose and amino acid and daily changes under different natural conditions of temperature and photoperiod in Gilthead Sea bream (*Sparus aurata*, L.). Fish Physiol Biochem 37: 583–592. 21174148 10.1007/s10695-010-9460-1

[ref27] Guardiola FA, Cuesta A, Esteban MÁ (2016) Using skin mucus to evaluate stress in gilthead seabream (*Sparus aurata* L.). Fish Shellfish Immunol 59: 323–330. 27818341 10.1016/j.fsi.2016.11.005

[ref28] Guelinckx J, Maes J, Van Den Driessche P, Geysen B, Dehairs F, Ollevier F (2007) Changes in δ13C and δ15N in different tissues of juvenile sand goby *Pomatoschistus minutus*: a laboratory diet-switch experiment. Mar Ecol Prog Ser 341: 205–215.

[ref29] Hesslein RH, Hallard KA, Ramlal P (1993) Replacement of sulfur, carbon, and nitrogen in tissue of growing broad whitefish (*Coregonus nasus*) in response to a change in diet traced by delta34S, delta13C, and delta15N. Can J Fish Aquat Sci 50: 2071–2076.

[ref30] Ibarz A, Ordónez-Grande B, Sanahuja I, Sánchez-Nunõ S, Fernández-Borras J, Blasco J, Fernández-Alacid L (2019) Using stable isotope analysis to study skin mucus exudation and renewal in fish. J Exp Biol 222. 10.1242/jeb.195925 30940672

[ref31] Ingram GA (1980) Substances involved in the natural resistance of fish to infection—a review. J Fish Biol 16: 23–60.

[ref32] Lazado CC, Skov PV (2019) Secretory proteins in the skin mucus of nile tilapia (*Oreochromis niloticus*) are modulated temporally by photoperiod and bacterial endotoxin cues. Fishes 4.

[ref33] Lee C-S (2015) Dietary Nutrients, Additives, and Fish Health. Wiley-Blackwell, Amsterdam, p. 384.

[ref34] Logan J, Haas H, Deegan L, Gaines E (2006) Turnover rates of nitrogen stable isotopes in the salt marsh mummichog, *Fundulus heteroclitus*, following a laboratory diet switch. Oecologia 147: 391–395. 16249895 10.1007/s00442-005-0277-z

[ref35] MacAvoy SE, Macko SA, Arneson LS (2005) Growth versus metabolic tissue replacement in mouse tissues determined by stable carbon and nitrogen isotope analysis. Can J Zool 83: 631–641.

[ref36] Martín-Perez M, Fernandez-Borras J, Ibarz A, Millan-Cubillo A, Felip O, De Oliveira E, Blasco J (2012) New insights into fish swimming: a proteomic and isotopic approach in gilthead sea bream. J Proteome Res 11: 3533–3547. 22681184 10.1021/pr3002832

[ref37] Martin-Perez M, Fernandez-borras J, Ibarz A, Felip O, Fontanillas R, Gutierrez J (2013) Naturally occurring stable isotopes reflect changes in protein. J Agric Food Chem 61: 8924–8933. 23947425 10.1021/jf402617h

[ref38] Martín-Pérez M, Fernández-Borràs J, Ibarz A, Felip O, Gutiérrez J, Blasco J (2011) Erratum: stable isotope analysis combined with metabolic indices discriminates between gilthead sea bream (*Sparus aurata*) fingerlings produced in various hatcheries. J Agric Food Chem 59: 11893. 10.1021/jf201670t 21838305

[ref39] Maruyama A, Yamada Y, Rusuwa B, Yuma M (2001) Change in stable nitrogen isotope ratio in the muscle tissue of a migratory goby, Rhinogobius sp., in a natural setting. Can J Fish Aquat Sci 58: 2125–2128.

[ref40] Maruyama A, Shinohara K, Sakurai M, Ohtsuka T, Rusuwa B (2015) Microhabitat variations in diatom composition and stable isotope ratios of the epilithic algae in Lake Malawi. Hydrobiologia 748: 161–169.

[ref41] Maruyama A, Tanahashi E, Hirayama T, Yonekura R (2017) A comparison of changes in stable isotope ratios in the epidermal mucus and muscle tissue of slow-growing adult catfish. Ecol Freshw Fish 26: 636–642.

[ref42] Montoya A, López-Olmeda JF, Garayzar ABS, Sánchez-Vázquez FJ (2010) Synchronization of daily rhythms of locomotor activity and plasma glucose, cortisol and thyroid hormones to feeding in gilthead seabream (*Sparus aurata*) under a light-dark cycle. Physiol Behav 101: 101–107. 20434474 10.1016/j.physbeh.2010.04.019

[ref43] Patel DM, Brinchmann MF (2017) Skin mucus proteins of lumpsucker (*Cyclopterus lumpus*). Biochem Biophys Rep 9: 217–225. 28956008 10.1016/j.bbrep.2016.12.016 PMC5614610

[ref44] Patel DM, Bhide K, Bhide M, Iversen MH, Brinchmann MF (2019) Proteomic and structural differences in lumpfish skin among the dorsal, caudal and ventral regions. Sci Rep 9: 1–13. 31061513 10.1038/s41598-019-43396-z PMC6502863

[ref45] Pickering AD (1974) The distribution of mucous cells in the epidermis of the brown trout *Salmo trutta* (L.) and the char *Salvelinus alpinus* (L.). J Fish Biol 6: 111–118.

[ref46] Pittman K, Pittman A, Karlson S, Cieplinska T, Sourd P, Redmond K, Ravnøy B, Sweetman E (2013) Body site matters: an evaluation and application of a novel histological methodology on the quantification of mucous cells in the skin of Atlantic salmon, *Salmo salar* L. J Fish Dis 36: 115–127. 23009125 10.1111/jfd.12002

[ref47] Pérez-Sánchez J, Terova G, Simó-Mirabet P, Rimoldi S, Folkedal O, Calduch-Giner JA, Olsen RE, Sitjà-Bobadilla A (2017) Skin mucus of gilthead sea bream (*Sparus aurata* l.). protein mapping and regulation in chronically stressed fish. Front Physiol 8: 1–18. 28210224 10.3389/fphys.2017.00034 PMC5288811

[ref48] Rajan B, Fernandes JMO, Caipang CMA, Kiron V, Rombout JHWM, Brinchmann MF (2011) Proteome reference map of the skin mucus of Atlantic cod (*Gadus morhua*) revealing immune competent molecules. Fish Shellfish Immunol 31: 224–231. 21609766 10.1016/j.fsi.2011.05.006

[ref49] Reyes-López FE, Vallejos-Vidal E, Ordóñez-Grande B, Sanahuja I, Sánchez-Nuño S, Fernández-Alacid L, Firmino J, Pavez L, Rodríguez L, Polo J et al. (2019) Searching integrated strategies for the evaluation of the physiological status in fish fed functional diets: the example of SDPP in gilthead sea bream (*Sparus aurata*). Fish Shellfish Immunol 91: 456.

[ref50] Roth J, Hobson K (2000) Stable carbon and nitrogen isotopic fractionation between diet and tissue of captive red fox: implications for dietary reconstruction. Can J Zool 78: 848–852.

[ref51] Sanahuja I, Ibarz A (2015) Skin mucus proteome of gilthead sea bream: a non-invasive method to screen for welfare indicators. Fish Shellfish Immunol 46: 426–435. 26134830 10.1016/j.fsi.2015.05.056

[ref52] Sanahuja I, Fernández-Alacid L, Sánchez-Nuño S, Ordóñez-Grande B, Ibarz A (2019a) Chronic cold stress alters the skin mucus interactome in a temperate fish model. Front Physiol 9: 1916. 30687126 10.3389/fphys.2018.01916 PMC6336924

[ref53] Sanahuja I, Fernández-Alacid L, Ordóñez-Grande B, Sánchez-Nuño S, Ramos A, Araujo RM, Ibarz A (2019b) Comparison of several non-specific skin mucus immune defences in three piscine species of aquaculture interest. Fish Shellfish Immunol 89: 428–436. 30978446 10.1016/j.fsi.2019.04.008

[ref54] Schindelin J, Rueden CT, Hiner MC, Eliceiri KW (2015) The ImageJ ecosystem: an open platform for biomedical image analysis. Mol Reprod Dev 82: 518–529. 26153368 10.1002/mrd.22489 PMC5428984

[ref55] Schwarcz HP, Schoeninger MJ (1991) Stable isotope analyses in human nutritional ecology. Am J Phys Anthropol 34: 283–321.

[ref56] Shephard KL (1993) Mucus on the epidermis of fish and its influence on drug delivery. Adv Drug Deliv Rev .

[ref57] Shephard KL (1994) Functions for fish mucus. Rev Fish Biol Fish 4: 401–429.

[ref58] Shigeta K, Tsuma S, Yonekura R, Kakamu H, Maruyama A (2017) Isotopic analysis of epidermal mucus in freshwater fishes can reveal short-time diet variations. Ecol Res 32: 643–652.

[ref59] Thomas SM, Crowther TW (2015) Predicting rates of isotopic turnover across the animal kingdom: a synthesis of existing data. J Anim Ecol 84: 861–870. 25482029 10.1111/1365-2656.12326

[ref60] Vander Zanden MJ, Clayton MK, Moody EK, Solomon CT, Weidel BC (2015) Stable isotope turnover and half-life in animal tissues: a literature synthesis. PLoS One 10: 1–16. 10.1371/journal.pone.0116182 PMC4321325 25635686

[ref61] Vatsos IN, Kotzamanis Y, Henry M, Angelidis P, Alexis MN (2010) Monitoring stress in fish by applying image analysis to their skin mucous cells. Eur J Histochem 54: 107–111. 10.4081/ejh.2010.e22 PMC3167306 20558343

[ref62] Winter ER, Nolan ET, Busst GMA, Britton JR (2019) Estimating stable isotope turnover rates of epidermal mucus and dorsal muscle for an omnivorous fish using a diet-switch experiment. Hydrobiologia 828: 245–258.

[ref63] Xia B, Gao QF, Dong SL, Wang F (2013) Carbon stable isotope turnover and fractionation in grass carp *Ctenopharyngodon idella* tissues. Aquat Biol 19: 207–216.

[ref64] Zaccone G, Kapoor BG, Fasulo S, Ainis L (2001) Structural, histochemical and functional aspects of the epidermis of fishes. Adv Mar Biol 40: 253–348.

